# Public wellbeing analytics framework using social media chatter data

**DOI:** 10.1007/s13278-022-00987-5

**Published:** 2022-11-03

**Authors:** Heba Ismail, M. Adel Serhani, Nada Hussien, Rawan Elabyad, Alramzana Navaz

**Affiliations:** 1 College of Engineering, Abu Dhabi University, Abu Dhabi, UAE; 2 College of IT, United Arab Emirates University, Al Ain, UAE

**Keywords:** Public wellbeing, Social media, Twitter, Emotion lexicon, Distant supervision, Sentence embeddings, Spatiotemporal analytics

## Abstract

Public wellbeing has always been crucial. Many governments around the globe prioritize the impact of their decisions on public wellbeing. In this paper, we propose an end-to-end public wellbeing analytics framework designed to predict the public’s wellbeing status and infer insights through the continuous analysis of social media content over several temporal events and across several locations. The proposed framework implements a novel distant supervision approach designed specifically to generate wellbeing-labeled datasets. In addition, it implements a wellbeing prediction model trained on contextualized sentence embeddings using BERT. Wellbeing predictions are visualized using several spatiotemporal analytics that can support decision-makers in gauging the impact of several government decisions and temporal events on the public, aiding in improving the decision-making process. Empirical experiments evaluate the effectiveness of the proposed distant supervision approach, the prediction model, and the utility of the produced analytics in gauging the public wellbeing status in a specific context.

## Introduction

The increase in complexity and pace of modern life worldwide has contributed to a higher percentage of wellbeing issues. Governments spend a fortune on supporting public wellbeing. According to the Global Burden of Disease (GBD) Study 2017, the global economic burden of mental diseases was estimated to be $ 99.5 million (Khaledi et al. 2019). Public wellbeing is undeniably affected by several life events. An evident occasion representing this significant impact is the outbreak of COVID-19 in March 2020. The unprecedented global outbreak of COVID-19 has caused governments worldwide to force several strict disease control measures (Li et al. 2020a, b). These control measures resulted in increased negative emotions in public, hindering the public’s wellbeing (Kydros et al. 2021; N et al. 2021). Even after the pandemic's peak, these negative consequences on public wellbeing still hold so far. In an attempt to assess public wellbeing, many governments have conducted several country-wide surveys to assess the public’s wellbeing (Allen et al. 2021; Shahriarirad et al. 2021; Son et al. 2020; Thapa et al. 2020). However, the public wellbeing surveys used are deficient in inferring a holistic insight into the public’s wellbeing due to the content limitation (i.e., intensive use of closed-ended questions), the subjectivity of the analysis, time limitations, geographic limitations, and other factors (Jones et al. 2008; Schwartz et al. 2014). This has necessitated a more holistic framework, adaptive to variable contexts and semantics, to assess the public wellbeing problems associated with various events. Social media platforms can give a representative and semantically-rich insight into the public’s wellbeing. During the pandemic and on different other occasions such as elections, new year, holiday seasons, or war, people find social media as a free platform to exchange opinions, express emotions and discuss related concerns (Dubey 2020). Twitter among other social media platforms is seen as the most popular and accessible social media platform for sharing public concerns (Kydros et al. 2021). In several studies, Twitter was considered a reliable source revealing wellbeing indicators (Boon-Itt & Skunkan 2020; De Choudhury et al. 2013). Therefore, this study targets Twitter owing to its popularity and accessibility; however, the proposed framework applies to other social media platforms.

To this end, we propose an end-to-end public wellbeing assessment framework that integrates semantically-rich sentence embeddings with predictive models to provide spatiotemporal, real-time analytics on public wellbeing using unstructured data found on social networking sites. Wellbeing indicators such as emotional orientation, the level of public interaction with positive and negative opinions, and emotional intensity relevant to specific events, in specific locations, and over variable timeframes will be analyzed in the proposed framework. Sentence embeddings are generated using the Pretrained Deep Bidirectional Transformer Network (BERT) (Devlin et al. 2019). Wellbeing training datasets in the proposed model are produced regularly using a novel distant supervision approach to provide context-relevant training data.

The proposed framework overcomes many challenges reported in the literature on automatic wellbeing assessment from the unstructured text found on social media. For instance, the proposed framework implements a novel heuristic-based, distant supervision method using an expert’s-verified wellbeing lexicon, providing a current and semantically relevant wellbeing training dataset. Wellbeing indicators in unstructured text vary depending on the linguistic context. User-generated unstructured text on social media differs over time, covering various topics of interest influenced by several life events. As such, historical training data used in previous research studies might not bear semantic relationships relevant to the current context, making them less valuable in training predictive models for a different timeframe (Almouzini et al. 2019; Chatterjee et al. 2019; Coppersmith et al. 2015; Scheuer et al. 2020).

In addition, manual annotation approaches commonly used in the literature on wellbeing assessment are time-consuming and inclined to subjectivity (Almouzini et al. 2019; Chatterjee et al. 2019; Coppersmith et al. 2015; Scheuer et al. 2020). Therefore, in this study, a wellbeing-specific distant supervision method is proposed to produce current training datasets bearing semantic relationships to emerging contexts. This alleviates the need for time-consuming manual annotation. Moreover, the proposed framework overcomes the feature engineering challenge inherent in the unstructured text stated by several studies in the field of wellbeing assessment (Almouzini et al. 2019; Chatterjee et al. 2019; Coppersmith et al. 2015; Scheuer et al. 2020) by regularly generating semantically-rich sentence embeddings using BERT from current social media content. In that sense, the proposed framework is adaptive to the changes in the semantic relationships and contextual features in the user-generated unstructured content, providing a more accurate assessment of wellbeing status. For further validation, we present a use case from the education context to demonstrate the utility of the proposed framework in inferring wellbeing insights related to COVID-19.

This paper is organized as follows: first, we review and summarize the literature on automatic wellbeing assessment from unstructured text, highlighting the several challenges addressed by other research studies, and then we describe the proposed framework and its main components. Afterward, we conduct a series of experiments to validate the proposed distant supervision method, the wellbeing predictions, and finally, the utility of the proposed framework in a particular context.

## Literature review

This section reviews related literature in the field of automated wellbeing assessment using social media content. We start by reviewing literature related to public wellbeing assessment highlighting limitations of existing research, and then we introduce the challenges related to feature engineering, contextual language models, and the inadequacy of training datasets for wellbeing assessment. The reviewed research studies are summarized in Table 1.

**Table 1:** Summary of relevant research works focused on analyzing wellbeing from Twitter content highlighting features, annotation method, and prediction accuracies

Work	Language	Method		Features						Ground Truth Annotation		
		Machine Learning	Deep Learning	BoW	Topics	Emotion Terms	Behavioural	Metadata	Embeddings	Expert	Survey	Self-declared
Tsugawa et al. (2013)	Japanese	Regression (Reg Coef 0.43)		✓							SDS	
Resnik et al. (2015)	English	Support Vector Regression Precision (62%-74%)		✓	LDA SLDA SNLDA	LIWC				✓		✓
Resnik et al. (2013)	English	Linear Regression Precision (43%-50%)			LDA	LIWC					Big-5 BDI	
Tsugawa et al. (2015)	Japanese	Support Vector Machines Accuracy (61%-66%)		✓	LDA	Japanese Lexicon	✓	✓			CES-D BDI	✓
De Choudhury et al. (2013)	English	Support Vector Machines Accuracy (70%)				LIWC	✓	✓			CES-D BDI	✓
Reece et al. (2016)	English	Random Forest–ROC (0.87–0.89)				LIWC A-NEW	✓	✓		✓	CES-D TSQ	✓
Almouzini et al. (2019)	Arabic	Multiple Classifiers Accuracy (55%–87%)		✓							CES-D PHQ-9	
Shetty et al. (2020)	English	Multiple Classifiers Accuracy (72%–76%)	LSTM & CNN Accuracy (93%–95%)	✓						NA	NA	NA
Husseini Orabi et al. (2018)	English	SVM –Accuracy (73%–77%)	CNN &RNN Accuracy (51%-87%)	✓					Word2Vec CBOW Skip-grams	✓		✓

### Analyzing the public wellbeing

Understanding the emotional status of the public across various instantaneous and ever-changing events is of utmost priority to all countries. Therefore, several studies investigated public wellbeing and mental health status through the automatic analysis of social media platforms or through surveying techniques. The reported studies on automatic assessment of public wellbeing using social media content focused mainly on emotions and sentiment analysis of user-generated text in specific locations. The most recent studies analyzed the influence of COVID-19 on public wellbeing in Iran, India, Norway, Sweden, the USA, and Canada (Bustos et al. 2022; Li et al. 2020a, b; Shahriarirad et al. 2021), China (Li et al. 2020a, b; Zhou et al. 2020), as well as Greek (Kydros et al. 2021). While the majority focused on predicting positive and negative sentiments, some studies analyzed distinct emotions such as Fear, Sadness, and Disgust. The reported studies do not conduct thorough spatiotemporal analysis and employ manually annotated datasets from previous contexts. On the other side, several studies analyzed public wellbeing through surveying techniques. For instance, Patrick et al. (2020) conducted a national survey from June 5 to 10, 2020, to assess the COVID-19 pandemic and mitigation efforts that affected the physical and emotional wellbeing of parents and children in the USA. However, the collected data was at a one-time point rather than longitudinally. Also, the survey results were biased since the respondents had higher socioeconomic status levels than non-respondents. Another study by VanderWeele et al. (2021) investigated the impact of the COVID-19 pandemic on physical and mental health from January to June 2020. The study employed a 15-min questionnaire, and the participants were selected using a stratified national sample of adults 18 and older within the USA. The study investigation was constrained by the sampling technique, the online assessment's requirement for internet access, the potential seasonality of respondents' wellbeing, and the potential for selection bias given that respondents in June were less likely to participate than in January. Krendl & Perry (2021) examined older adults’ mental health and social wellbeing in a short time to measure social isolation due to COVID-19. Between April and May 2020, around 93 older adults in the USA took place on phone interviews conducted by the authors. Moreover, a study by Wang et et al. (2020) analyzed the mental health outcomes associated with the covid-19 pandemic. They used online surveys of the Chinese population with a snowball sampling design. The survey took place between January and February 2, 2020, with a total of 1210 respondents from 194 cities in China. Notwithstanding, the selection bias resulted from an oversampling of a specific network of peers. Therefore, the finding was less applicable to the overall population, especially those with lower levels of education. Therefore, to our best knowledge, the existing studies in the field of public wellbeing analysis are usually designed for a specific timeframe, location, or sample, or rely on manually annotated data from different contexts. None of the reported studies provided an end-to-end framework that starts with generating a wellbeing-specific labeled dataset leading to inferring spatiotemporal insights across variable timeframes and geo-locations.

### Feature engineering challenges in machine learning-based methods for wellbeing assessment

Several research efforts in automated wellbeing assessment on social media focused on building classical machine learning predictive models, which relied on “features” (Resnik et al. 2013, 2015; Tausczik & Pennebaker 2010). These studies focused mainly on linguistic features extracted from user-generated text. For instance, Tsugawa et al. (2013) utilized frequency bag-of-word (BoW), while Resnik et al. (2015), and Armstrong (Resnik et al. 2013), used various combinations of topic models. In addition, some studies tried to improve on results obtained from linguistic features by extracting terms with emotional sentiment based on emotions’ lexicons, such as the Linguistic Inquiry Word Count (LIWC) Lexicon (Tausczik & Pennebaker 2010). For example, Schwartz et al. (2014) utilized some emotion lexicons, including LIWC, to generate several feature vectors. A major challenge inherent in using linguistic features of all types is the very high dimensionality of the feature space, which usually leads to sparse datasets. Therefore, various features selection techniques have been applied to obtain the most prevalent features, such as correlation analysis (Tsugawa et al. 2013), attempting different classification thresholds (Resnik et al. 2015), or even involving a clinical psychologist to manually evaluate the extracted features (Resnik et al. 2013), a task which is expensive and time-consuming.

Besides the feature selection challenge, reported prediction accuracy, relying barely on linguistic features, was not very promising, as depicted in Table 1. Consequently, several studies attempted different behavioral and metadata features, which were argued in the literature to be more prevalent in wellbeing issues. For instance, Tsugawa et al. (2015) used, in addition to frequency bag-of-word (BoW) and Latent Dirichlet Allocation (LDA) topics, the ratio of positive-affect and negative-affect words based on a Japanese affect dictionary (Inui et al. 2013) Furthermore, they analyzed behavioral features such as tweet time, hashtags, and URLs indicating some symptoms of depression such as insomnia or isolation. Choudhury et al. (2013) explored even more sophisticated features by looking at the use of depression-related vocabulary based on terms extracted from Yahoo Answers under the “Wellbeing” Category. They also counted the frequency of names of antidepressants using a “list of antidepressants” from the Wikipedia page and extracted egocentric features based on social network analysis; (a) node properties, (b) dyadic properties, and (c) network properties. Reece et al. (2016) explored the combination of multiple emotion lexicons with a more comprehensive set of behavioral and metadata features in addition to time series features. Even though prediction results improved slightly with the addition of behavioral and metadata features, complexity increased too, adding more overhead and making these methods very expensive and time-consuming.

Moreover, to accurately extract and validate all these features, one must have adequate knowledge relevant to wellbeing research making these approaches unsuitable for continuous public wellbeing assessments. Moreover, latent semantics and contextual linguistic signs are key indicators of wellbeing and other emotional states which cannot be ignored. Nevertheless, features extracted from text, or other types of behavioral features, lack context. Consequently, it has become imperative to employ efficient prediction models that do not require explicit feature extraction or identification and modeling of contextual attributes.

### Contextual language models’ limitations in deep learning-based wellbeing assessment methods

Deep Learning (DL) algorithms integrating automatic feature representation have made impressive advances in the field of natural language processing (NLP), alleviating the inherent limitations in classical machine learning algorithms that depend on hand-crafted features (Young et al. 2018). For instance, Shetty et al. (2020) used raw twitter data with two Deep Learning algorithms; long short-term memory (LSTM) and sequential Convolutional Neural Network (CNN). They compared results with multiple machine learning classifiers trained on TF-IDF and frequency BoWs. Results of Deep Learning classifiers surpassed those of machine learning classifiers reducing the overhead associated with feature extraction and selection. Moreover, Orabi et al. (2018) investigated the use of multiple word-embeddings. They used three word embeddings; Word2Vec, CBOW, and Skip-grams, with CNN and Recurrent Neural Network (RNN), compared to SVM trained on TF-IDF BoW. Results obtained from DL predictive models suggest that DL models trained on word embeddings can outperform the prediction accuracy of classical machine learning and DL models trained on discrete word vectors for wellbeing assessment. Word vectors overcome some challenges inherent in features engineering highlighted earlier. However, they suffer from three main limitations. Word vectors are context-independent, i.e., (1) do not reflect contextual relationships among words, (2) do not account for unknown words, i.e., they do not support transfer-learning to a model using a different vocabulary of the same size, and (3) the size of vectors depends on the size of vocabulary used, which can be huge, resulting in the curse of dimensionality problem. Nevertheless, context can reveal useful wellbeing indicators. Hence, in this research, we intend to address the feature engineering challenges explained above and the context-aware challenge by implementing the Pretrained Deep Bidirectional Transformer Network (BERT) to regularly generate fully contextualized sentence embeddings relevant to different life events reflecting a more relevant representation of wellbeing linguistic markers.

### Inadequacy of existing labelled training data for wellbeing

Likewise, the feature engineering and context modeling challenges highlighted earlier, acquiring accurate and representative labeled training data is a persisting challenge. Wellbeing datasets obtained from social media platforms already exist. However, they are specific to previous timeframes and reflect different contexts and topics of interest. For instance, there exist twitter datasets focusing on public wellbeing post-COVID-19 (Kleinberg et al. 2020). Moreover, some datasets were collected after explicitly asking participants to express and reflect on a particular topic, for example, (Scheuer et al. 2020). Furthermore, some are solely focused on emotion detection only (Chatterjee et al. 2019) or diagnosing certain mental health disorders such as depression (Almouzini et al. 2019) and post-traumatic-stress disorder (PTSD), depression, bipolar disorder, and seasonal affective disorder (SAD) (Coppersmith et al. 2015).

Nevertheless, as illustrated in Table 1, annotation approaches in the field of wellbeing assessment classically relied on manual annotation by (i) wellbeing experts (Resnik et al. 2015), (ii) psychometric questionnaires (e.g., CES-D & BDI) through crowdsourcing (Tsugawa et al. 2015), (De Choudhury et al. 2013), or (iii) self-reporting (Guntuku et al. 2017). These labeling methods cannot be used under different circumstances in various contexts. We summarize the inherent limitations hereafter:

1. They are expensive, time-consuming, and rely solely on manual annotation. With the ever-evolving number of Tweets related to wellbeing, we need a reliable automated labeling approach capable of annotating current, real-time data with minimal manual intervention to reflect the specific semantics relevant to this data.
2. Available wellbeing training datasets focus on patients diagnosed with a mental disorder. In previous research, identified users' tweets are usually tracked for one year (De Choudhury et al. 2013; Tsugawa et al. 2015). We are looking for early indicators to understand public well-being status and support people early on before developing mental disorders.
3. Existing labeled data for wellbeing are outdated and collected from different contexts. We need timely labeled data to analyze emotional triggers relevant to the public’s wellbeing for different spatiotemporal events and provide more relevant support.

## Methods

**Fig. 1:**
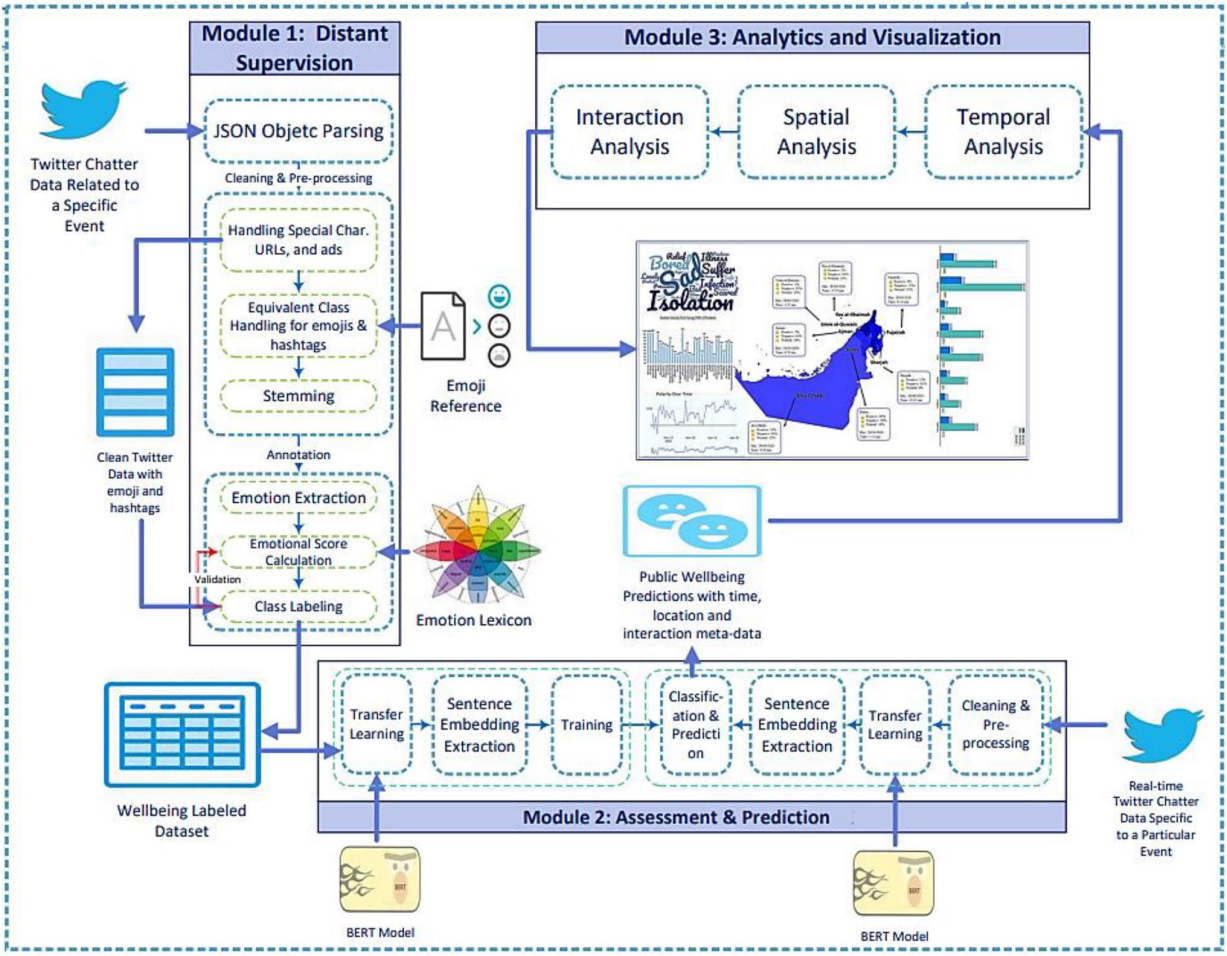
Public wellbeing analytics framework

To address the above-mentioned research challenges and achieve the research aims, we propose a framework composed of three main modules as illustrated in Fig. 1: (1) Distant Supervision, (2) Assessment and Prediction, and (3) Analytics and Visualization. The three modules collaborate to infer real-time insights into public wellbeing indicators to support decision-makers plan, design, and implement appropriate interventions. In subsequent sections, we briefly describe the tasks carried out in each module.

### Module 1: distant supervision

This module is designed to generate current wellbeing-related labeled datasets based on distant supervision. The context changes with the continuously changing situations. Survey-based (De Choudhury et al. 2013; Tsugawa et al. 2015) and expert-based (Resnik et al. 2015) approaches, commonly used in the field of automatic wellbeing assessment, are not practical nor ideal solutions for rapidly changing contexts. Also, historical ground truth benchmark data generated from other contexts might not bear semantics and topics related to new and emerging situations, influencing individuals’ emotional states. This, in turn, hinders the accuracy of Predictions (Li et al. 2020a, b). Hence, we define and validate a fully automated, distant supervision approach suitable to generate up-to-date labeled data for any given situation or context based on verified emotion lexicons and expert-verified heuristics. This module's first, anonymized and representative, raw Twitter chatter data is hydrated in JSON format. Then, several subprocesses are carried out to ensure accurate annotation of data. These are: (1) parsing JSON objects’ attributes required for wellbeing prediction as well as for analytics modules, (2) cleaning and pre-processing tweet’s text to prepare it for annotation, and then (3) annotating the cleaned and pre-processed tweets based on emotional lexicons such that a total emotional score is calculated based on a predefined function. Hereafter, we briefly describe each one of these three sub-processes.

#### Parsing JSON objects

All Twitter APIs used to retrieve Twitter data encode this data using JavaScript Object Notation (JSON). JSON is based on key-value pairs with named attributes and associated values. These attributes and their state are used to describe objects. Twitter defines the number of JSON objects for each account. We focus primarily on the “Tweet Object” and the “User Object” to extract attributes such as ‘*text,’* ‘*place,’* ‘*hashtag,’* ‘*favorite_count*,’ and ‘*retweet_count*’. The “Text” attribute is primarily used to retrieve the user’s generated text and assess the use of negative language in addition to other insights. Other attributes are essential for the Analytics Module.

#### Cleaning and pre-processing tweet’s text

Twitter is full of peculiarities; on the other hand, these special characters, emojis, and hashtags might reveal valuable semantics relevant to users’ wellbeing, as indicated by several studies, e.g. (Alvarez-Jimenez et al. 2019). Click or tap here to enter text. Hence, we conduct pre-processing tasks such as tokenization, stemming, and sentiment replacement in two phases. Initially, we generate a clean dataset (i.e., tokenized and stemmed) with semantic replacements of emojis to enrich the tweet’s sentiment and enhance the accuracy of distant supervision. The Unicode of an emoji is searched, and the literal description is retrieved. From the literal description, words with emotional orientation are searched, such as ‘love’, ‘happy’, smile’…etc. Then, the emoji is replaced with its equivalent emotional literal. Then, we reflect the annotation on a more natural-looking dataset without sentiment replacement to generate more representative sentence embedding in the next module.

#### Annotation-based on distant supervision

**Fig. 2:**
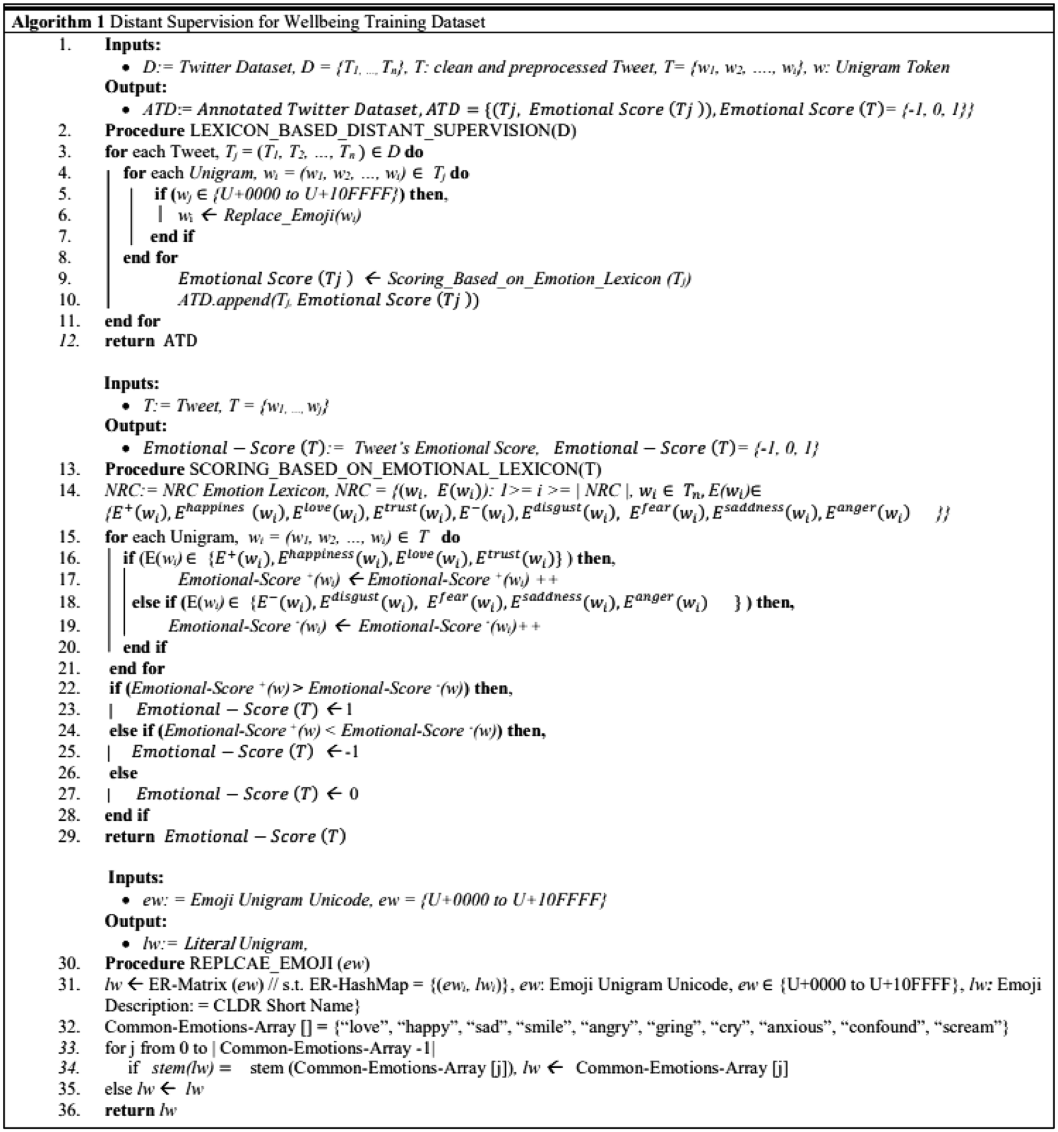
Distant supervision algorithm for wellbeing dataset

At this stage, the clean text is annotated based on the NRC emotion lexicon (Mohammad & Turney 2013) and Formula (1) following the Distant Supervision Algorithm illustrated in Fig. 2. The proposed lexicon-based annotation approach is different from previous methods as it focuses specifically on emotional terms which are considered representative of human mental wellbeing. The proposed approach does not consider only negative or positive words; rather, it focuses on the emotional orientation of the tweet. Therefore, terms with emotional orientation, as defined in the NRC lexicon, are searched in the tweet, and an emotional score, $Emotional-Score \left(T\right),$ is calculated accordingly, indicating the emotional orientation of the full tweet’s text. As such, a tweet, *T*, is made up of unigrams, *w*, such that *T* = *{w*_*1*_*, w*_*2*_*, …., w*_*i*_*}.* Each unigram may assume a negative or a positive emotional score or may be associated with a distinct emotion such as sadness, anger, happiness, trust, etc., as defined in the NRC lexicon. To account for all negative wellbeing statuses, we aggregate the scores of ‘disgust’, ‘fear’, ‘sadness’, and ‘anger’ with the ‘negative’ emotion score. In addition, we aggregate the scores of ‘happiness, ‘love’, and ‘trust’ with the ‘positive’ emotional score to account for the positive wellbeing status as explained in Eq. (1). Finally, if the sum of the negative emotional score is greater in a particular tweet than the sum of positive emotional scores, a negative label is assigned, and vice versa. Snowball stemming is applied to ensure the matching of all morphological variations of n-grams with related emotional terms in the lexicon. Neutral labels are assigned to tweets with equal emotional scores. The two procedures accomplishing these steps: *Lexicon_Based_Distant_Supervision(D)* and *Scoring_Based_On_Emotioal_Lexicon(T)* in Algorithm-1- are illustrated in Fig. 2. Given that Previous studies indicated that emojis could be used as wellbeing indicators conveying various emotional states (Alvarez-Jimenez et al. 2019), emojis’ are replaced with their emotional equivalent based on an emotion replacement matrix, *ER-HashMap*. *ER-HashMap* maps the emoji’s Unicode, *ew*, to its corresponding CLDR literal description, *el*. This task is performed by the *ReplaceEmoji(ew)* procedure in Algorithm-1- illustrated in Fig. 2. Such that if the emoji’s literal description, *el*, contains anything related to the ten explicit emotional terms as defined in the *Common-Emotions-Array*, line 32 in Algorithm-1-, then the explicit emotional term is used instead; otherwise, the literal description of the emoji is retrieved to be evaluated using the NRC lexicon. The proposed annotation algorithm is described in Fig. 2.

1 $$\begin{gathered} Emotional - Score \left( T \right) = \hfill \\ \left\{ {\begin{array}{*{20}c} { - 1 \,if \mathop \sum \limits_{i = 1}^{n} |E^{ + } \left( {w_{i} } \right) + E^{happiness} \left( {w_{i} } \right) + E^{love} \left( {w_{i} } \right) + E^{trust} \left( {w_{i} } \right)|\left\langle { \mathop \sum \limits_{i = 1}^{n} |E^{ - } \left( {w_{i} } \right) + E^{disgust} \left( {w_{i} } \right) + E^{fear} \left( {w_{i} } \right) + E^{saddness} \left( {w_{i} } \right) + E^{anger} \left( {w_{i} } \right)} \right|} \\ {1\, if \mathop \sum \limits_{i = 1}^{n} |E^{ + } \left( {w_{i} } \right) + E^{happiness} \left( {w_{i} } \right) + E^{love} \left( {w_{i} } \right) + E^{trust} \left( {w_{i} } \right)| > \mathop \sum \limits_{i = 1}^{n} |E^{ - } \left( {w_{i} } \right) + E^{disgust} \left( {w_{i} } \right) + E^{fear} \left( {w_{i} } \right) + E^{saddness} \left( {w_{i} } \right) + E^{anger} \left( {w_{i} } \right)|} \\ {0\, if \mathop \sum \limits_{i = 1}^{n} |E^{ + } \left( {w_{i} } \right) + E^{happiness} \left( {w_{i} } \right) + E^{love} \left( {w_{i} } \right) + E^{trust} \left( {w_{i} } \right)\left| { = \mathop \sum \limits_{i = 1}^{n} |E^{ - } \left( {w_{i} } \right) + E^{disgust} \left( {w_{i} } \right) + E^{fear} \left( {w_{i} } \right) + E^{saddness} \left( {w_{i} } \right) + E^{anger} \left( {w_{i} } \right)} \right|} \\ \end{array} } \right. \hfill \\ \end{gathered}$$

### Module 2: assessment and prediction

**Fig. 3:**
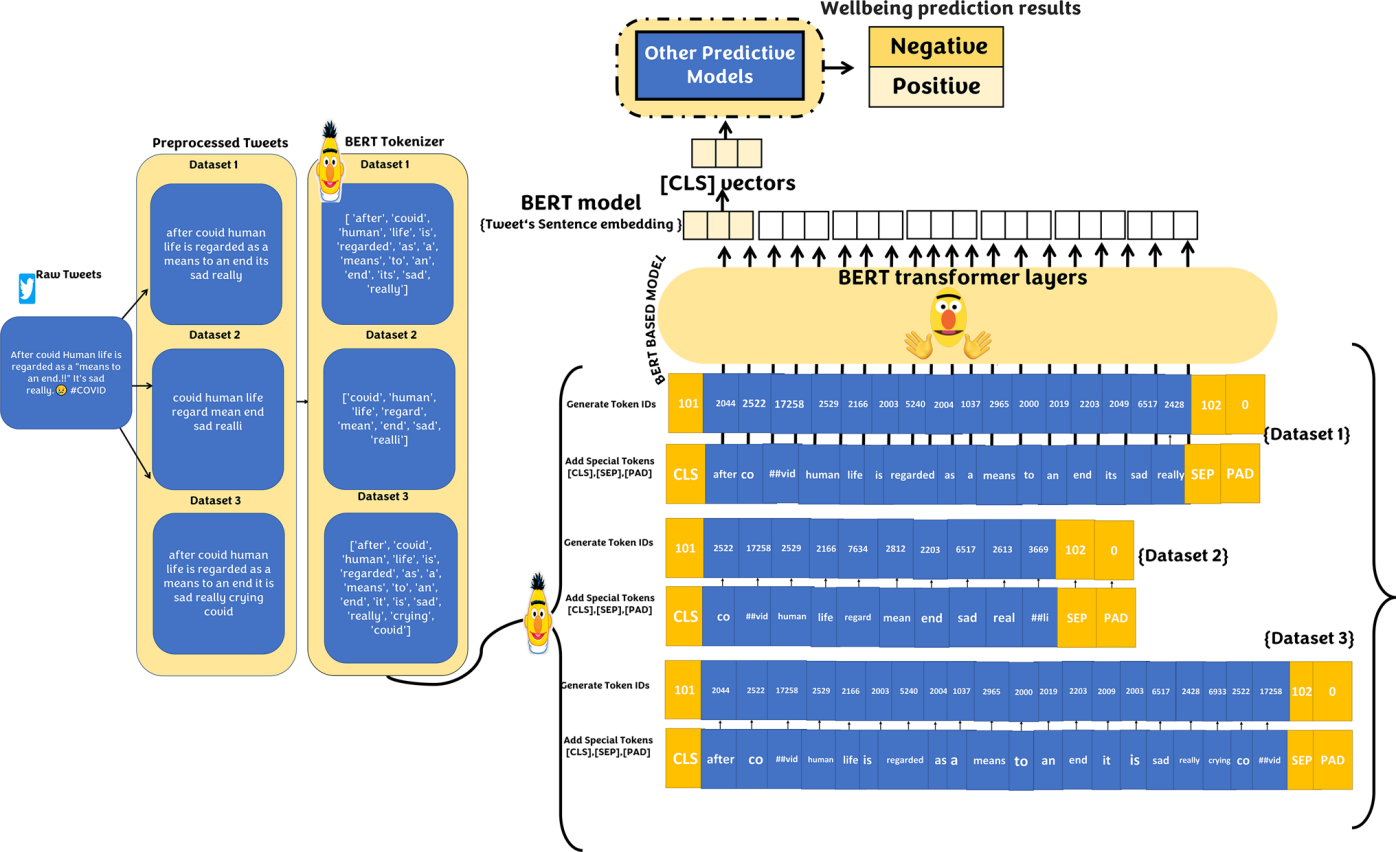
BERT-based context-specific sentence embedding

Several studies proved that people experiencing wellbeing issues tend to use negative language (De Choudhury et al. 2013; Park et al. 2015; Tsugawa et al. 2015); hence, we aim to evaluate public wellbeing through the use of negative language. In this module, first, real-time Twitter chatter data is retrieved and cleaned using similar pre-processing tasks explained in Module-1-. Then, the BERT model is used to generate rich contextual sentence embedding for the new chatter data and the labelled training data generated in Module-1-. Second, using the labelled sentence embeddings, several classifiers are trained to predict, in real-time, whether a user is experiencing wellbeing challenges or not. We use BERT_BASE_ (*L* = 12, *H* = 768, *A* = 12, Total Parameters = 110 M), where L is the number of layers, the hidden size as H, and the number of self-attention heads as A. We consider a tweet as a sentence, i.e., a sequence of tokens composed of one or multiple sentences. More formally, let us consider a tweet as$X=\{{x}_{1}, \dots , {x}_{n}\}$, where *n* denotes the length of the Tweet such that$\left|X\right|=n$. BERT adds two special tokens to the input sequence. The first token of every sequence is always a special classification token ([CLS]), and the last token is a separation token ([SEP]). Hence, in our problem, a Tweet is defined as$X=\{\mathrm{CLS}, {x}_{1}, \dots , {x}_{n },\mathrm{ SEP}\}$. BERT uses the final hidden state corresponding to ([CLS]) token as the aggregate sequence representation for classification tasks. Therefore, the output corresponding to ([CLS]) token can be thought of as an embedding for the entire sentence (i.e., tweet). We obtain the sentence embedding for all the tweets in the labelled training dataset as well as in the real-time chatter data by extracting the final vector output of this token. The labelled sentence embeddings are used as the feature set to train several classifiers. After that, the learned model is used to predict the wellbeing indicators in the new unlabelled real-time Twitter chatter data. Figure 3 illustrates how context-specific sentence embeddings for the tweets are generated using BERT model for the three experimental datasets listed in Sect. 4.2.1. As can be seen in Figure 3, for tweets' (i.e., sentences') wellbeing classification, we’re only interested in the first vector of the BERT’s output associated with the [CLS] token. That is because BERT is trained to encapsulate a sentence-wide sense of the output at the first position (i.e., at the CLS token) producing a contextual sentence embedding of the input. These contextual sentence embeddings are then used to train other predictive models to produce wellbeing predictions.

### Module 3: analytics and visualization

This module aims to assess and visualize the variability in the emotional status of the public through their Tweets over time across several locations based on the predictions generated in Module#2 and the associated meta-data. After conducting spatiotemporal analysis, and interaction analysis, we can measure the variability of public wellbeing indicators at different times of the day, over several time events, and across several cities aiding decision-makers in planning necessary intervention plans. For instance, the public reaction to schools’ closure might differ in emotional intensity from one city to another. By analyzing the variations in the intensity of public emotion relevant to different temporal events in several locations, the decision-makers can make more well-informed decisions. In addition, analyzing public emotions over several time intervals in the day provides crucial evidence of public wellbeing. Statistics show that nighttime online activity is a known characteristic of individuals susceptible to wellbeing issues, as highlighted and validated in (De Choudhury et al. 2013; Lustberg & Reynolds 2000). Hence, an increased nighttime Twitter activity can indicate the rise of wellbeing issues during the pandemic, besides other linguistic indicators.

Moreover, interaction analysis measures users’ responses to negative tweets after analyzing “retweet” and “favorite” activities. It is prevalent on social media platforms to have people navigating and surfing but never posting content. The interaction of this group of the public can give insight into their wellbeing status. As such, people interacting with negative Tweets are more likely to be experiencing negative emotions and vice versa. To perform temporal and spatial analysis, metadata is retrieved from Twitter JSON object indicating cities in the country, interactions in the form of “Retweets” or “Likes”, as well as the time-related attributes. Therefore, several spatiotemporal and linguistic visualizations, such as sentiment maps, wordclouds, and sentiment bars, are used to provide insight into the public’s emotional status.

## Experimental work and results

In this section, we demonstrate experimental results as follows:

Section 4.1 validates the proposed distant supervision approach to generate a wellbeing-related training dataset from unstructured text.

Section 4.2 validates the proposed BERT-based wellbeing prediction model.

Section 4.3 demonstrates how wellbeing predictions from Twitter can reveal decision-support critical insights through a case study related to the education sector.

### Validating distant supervision method

To validate the proposed distant supervision method for wellbeing dataset labeling, we use the first published COVID-19 Twitter dataset, collected on January 22, 2020 (Chen et al. 2020). This data is selected as it contains intensive wellbeing indicators considering the context of the COVID-19 outbreak. Twitter chatter data is preprocessed as explained in 3.1.1 and 3.1.2, and an overall emotional score of the tweet is calculated as explained in Fig. 2. Table 2 shows examples of tweets before and after pre-processing.

**Table 2:** Tweets before and after preprocessing and emoji replacement

Original Tweet	Processed tweet	Annotation
We can heal the Earth https://t.co/RwrL0BR1Vf	we can heal the earth love	Positive
Please retweet. Yes Trump said worries about cornov is the new hoax . https://t.co/SsKL9Wuq4L	please retweet yes trump said worries about cornov is the new hoax angry	Negative

Next, manual validation is conducted on two annotated datasets by human expert annotators. Each dataset contains 16,797 tweets. Using stratified sampling, two representative samples of 580 tweets are selected from each dataset to ensure balanced examples proportional to each class label with a 95% confidence level and confidence interval of 4. We evaluated the agreement between experts and the method’s annotation using Kappa interrater agreement test. Kappa scores were 0.85 and 0.81 for the first and second samples, respectively. These Kappa scores indicate good agreement between the human rater and the proposed annotation method rating. Minor discrepancies between the proposed method’s annotation and the expert’s annotation were identified due to the extensive use of informal language on Twitter. For example, cases of sarcasm and irony are not perfectly captured by the distant supervision approach, especially for concise tweets. However, those were minor cases as typically, users would use irony in conjunction with some representative emojis which are handled in our approach. In addition, negation is not captured in distant supervision; the method does not consider “I am not happy” as a negative sentence because it contains the word ‘happy’ which has a positive emotional sentiment. However, in psychology, a person saying that they are "happy" or "not happy" is still demonstrating a focus on positivity as a dimension of thought. They could have just as easily said "sad" or "angry", but it is psychologically telling that they are thinking in terms of "happiness" (Pennebaker et al. 2003; Tausczik & Pennebaker 2010). This, in turn, supports the validity of the proposed distant supervision approach despite the few discrepancies caused by negation or sarcasm.

### Validating wellbeing predictions with BERT-based sentence embeddings

In this section, we validate the proposed approach for wellbeing predictions using BERT as a language model to provide rich contextualized sentence embeddings in conjunction with several classifiers. The purpose of these experiments is to prove that BERT as a language model gives more accurate predictions, to select the best classifier to be used for online predictions with Twitter data, as well as to select the best pre-processing tasks that would generate the highest prediction results.

Using distant supervision, explained in the previous section, an evaluation wellbeing dataset is generated. We consider original Tweets only, i.e., retweets and Quoted Tweets are omitted from this dataset. Also, repeated Tweets are removed. Hence the evaluation dataset contains unique Tweets collected during March 2020 upon declaring COVID-19 as a world pandemic as it is expected to be rich in wellbeing indicators. Statistics of the evaluation dataset after labelling using the proposed distant supervision method are summarized in Table 3. We also omit neutral Tweets as we are focusing on the positive and negative dimensions of thoughts.

**Table 3:** Statistics of evaluation dataset

Data	Size
Total	10,888
Negative tweets	9369
Positive tweets	1519

#### Pre-processing of the evaluation dataset

To identify the best pre-processing required for the assessment module, three versions of the evaluation dataset are generated; each had undergone different pre-processing tasks, as follows:

1. Dataset-1-: A dataset cleaned from URLs, mentions, hashtags, emoji, and special characters without any constraint on character length and stopwords are kept, i.e., Tweets are kept as natural sentences without abnormal tokens.
2. Dataset-2-**:** A dataset with similar pre-processing applied to Dataset-1; in addition, stopwords are removed, and the minimum character length is set to two characters. This is the typical pre-processing done in most of the sentiment and emotion analysis tasks (Ismail et al. 2018).
3. Dataset-3-: A dataset with similar pre-processing applied to Dataset-1, except that emojis are kept and replaced with their emotional equivalent, as illustrated in Table 2. Besides, abbreviated verbs are transformed into full form. Also, hashtags are kept, but the ‘#’ special character is removed. This is done to ensure that all emotions conveyed by a Twitter user are kept in the text. A task that is commonly done in emotion analysis.

#### Baselines with different feature vector models

To compare the performance of the classification model trained on sentence embeddings using BERT with other feature representations, we generate three baselines with different feature vector representations, commonly used in the literature on wellbeing automatic assessment (Husseini Orabi et al. 2018; Resnik et al. 2015; Tsugawa et al. 2015), as follows:

1. Baseline-1-: Term frequency inverse document frequency (TF-IDF) BoW features.
2. Baseline-2-: NRC emotion lexicon features are populated with frequencies.
3. Baseline-3-: Word2Vec, word embeddings’ features.

#### Experiments and results

For wellbeing’s prediction evaluation, support vector machines (SVM), K-nearest Neighbors (KNN), Logistic Regression (LR), and Decision Trees (DT), classifiers are trained on the full dataset using the different variants of the evaluation dataset explained earlier (i.e., Dataset-1-, Dataset-2-, Dataset-3-) with three different feature vector models. The predictive models are evaluated with stratified twofold cross-validation.

As illustrated in Table 3, the class distribution of the evaluation dataset is considerably skewed towards the negative class, which is caused by the increased negative feelings evolving post the COVID-19 outbreak during which the evaluation data was collected. This imbalanced class distribution is known to generate inaccurate classification results during evaluation and can lead to model overfitting problems during the training phase (Branco et al. 2015; Galar et al. 2012; Powers 2015). Hence, stratified cross-validation is used to avoid overfitting and ensure each class is approximately equally represented across each test fold proportionally to class distribution in the full dataset.

In addition, to accurately evaluate the quality for classification despite class skewness, precision and F-measures are selected to evaluate the classification quality (Liu et al. 2009). Precision indicates how well a classification model identifies positive classes (i.e., the majority class, and in our dataset, it represents tweets with negative emotions). We consider this as an essential quality criterion in the selected classification model. That is because we aim to specifically identify users susceptible to experiencing wellbeing challenges. On the other hand, the F-measure, i.e., the weighted average of Precision and Recall, is used to evaluate the classification balance between specificity and completeness. The higher the F-measure in this problem, the more balance between positive cases detected and the total number of positive cases in the dataset.

Looking at the classification results in Tables 4, 5 and 6, we can see that sentence embedding features, based on BERT, consistently generate highly precise results (between 0.81 and 0.87) using multiple classifiers trained on dataset-1-, i.e., more natural-looking text, and dataset-3-, i.e., with emotion replacement, as opposed to all other feature models. In contrast, feature vector models commonly used in the literature of wellbeing assessment, i.e., BoW and emotion features, generate lower precision values using all the evaluation datasets. This, in turn, supports using BERT for contextualized feature representation, especially with the possibility of using less processed text to get highly precise results. As such, this would work well with real-time data as we are in favor of reducing pre-processing overhead and predicting public wellbeing from the raw text as much as possible. BERT-based features also generate a good balance between positively detected cases out of all positive cases in the dataset as depicted by the F-measure (between 0.84 and 0.88) using dataset-1- and dataset-3- as opposed to all other feature vector models.

**Table 4:** Classification results for dataset-1

Features	Measures	LR	KNN	SVM	DT
BERT	Precision	0.81	0.86	0.87	0.84
	F-measure	0.85	0.87	0.88	0.84
BoW	Precision	0.7	0.67	0.87	0.61
	F-measure	0.47	0.56	0.54	0.61
NRC	Precision	0.45	0.47	0.45	0.47
	F-measure	0.47	0.47	0.47	0.47
Word2Vec	Precision	0.64	0.71	0.63	0.60
	F-measure	0.62	0.54	0.62	0.60

**Table 5:** Classification results for dataset-2

Features	Measures	LR	KNN	SVM	DT
BERT	Precision	0.74	0.83	0.84	0.8
	F-measure	0.8	0.83	0.85	0.8
BoW	Precision	0.68	0.63	0.80	0.58
	F-measure	0.46	0.57	0.56	0.58
NRC	Precision	0.43	0.56	0.43	0.53
	F-measure	0.46	0.51	0.46	0.47
Word2Vec	Precision	0.7	0.81	0.69	0.60
	F-measure	0.69	0.52	0.68	0.60

**Table 6:** Classification results for dataset-3

Features	Measures	LR	KNN	SVM	DT
BERT	Precision	0.81	0.87	0.87	0.85
	F-measure	0.85	0.87	0.88	0.84
BoW	Precision	0.7	0.67	0.87	0.61
	F-measure	0.47	0.56	0.53	0.61
NRC	Precision	0.45	0.45	0.45	0.47
	F-measure	0.47	0.47	0.47	0.47
Word2Vec	Precision	0.64	0.78	0.64	0.57
	F-measure	0.62	0.52	0.62	0.57

Interestingly, classification quality slightly drops when training classifiers with BERT-based features using dataset-2-, i.e., without stopwords and short tokens. Precision reaches its minimum value of 0.74 with LR when using dataset-2- with BERT. This is because BERT is trained on natural text, including stopwords, which makes it more powerful in understanding the full context. Even though word embeddings are known to generate rich semantics, i.e., adding context to words, word2vec feature vectors generated very low precision values (between 0.57 and 0.81) when compared with BERT's.

**Fig. 4:**
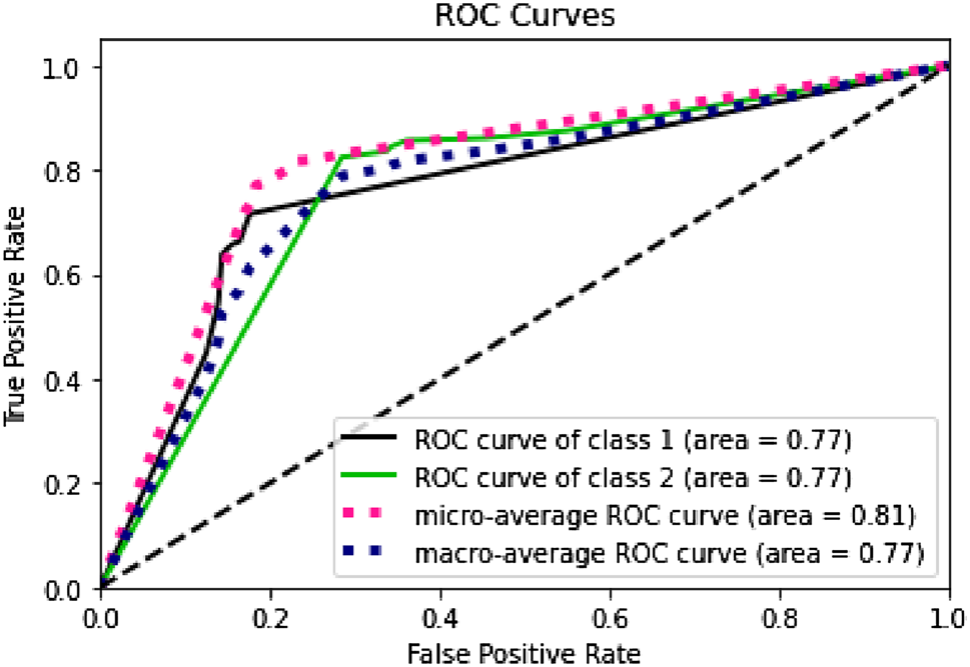
Decision tree

**Fig. 5:**
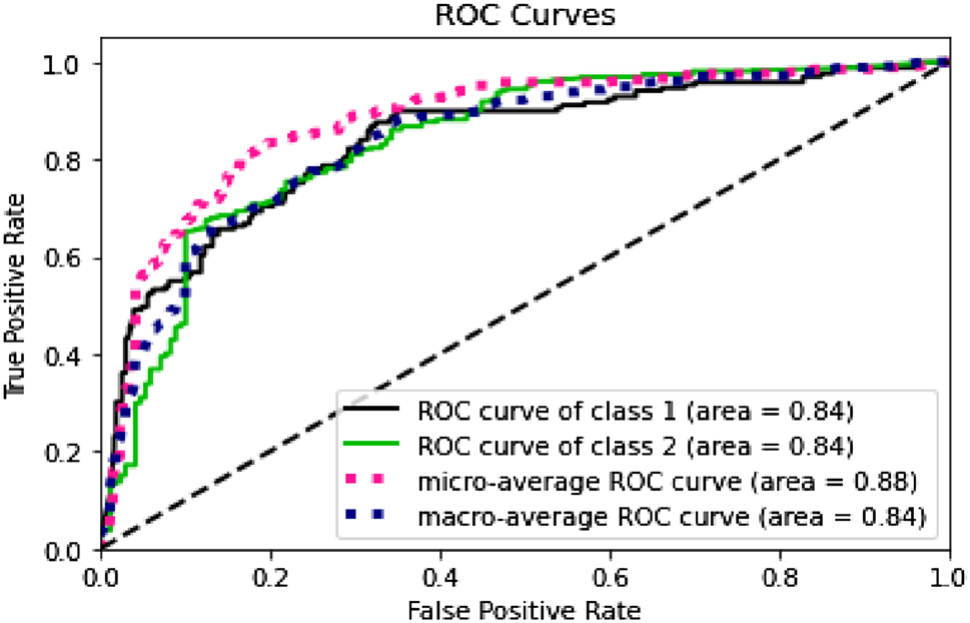
SVM

**Fig. 6:**
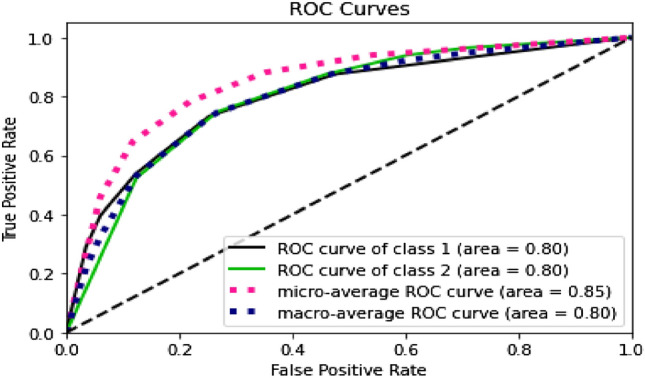
KNN

**Fig. 7:**
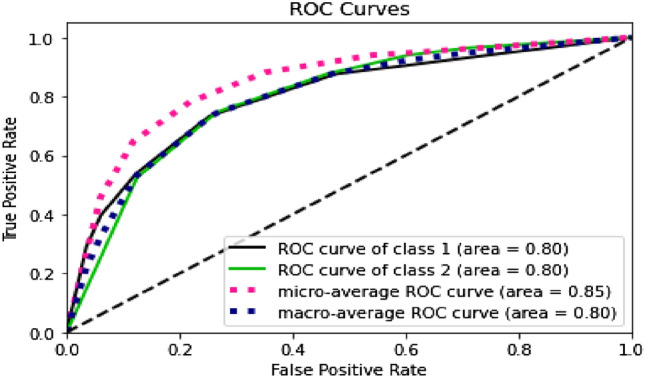
LR

Furthermore, by looking at the ROC curves in Figs. 4, 5, 6 and 7, we can see that the prediction models trained on BERT sentence embeddings as feature vectors based on Dataset-3-had consistently generated high accuracies with AUC ranging between 0.77 and 0.85 with the four classifiers (i.e., DT, SVM, KNN, and LR) for both classes. This in turn surpasses all reported prediction results in the literature of automatic wellbeing assessment based on Twitter dataset, as summarized in Table 1. We can further see that the prediction accuracy for both classes (i.e., the negative class and positive class) are, to some extent, comparable despite the imbalanced distribution of class labels. In addition, considering the micro average of the ROC Curve for both classes, the AUC ranges between 0.81 to 0.89 with the four classifiers (i.e., DT, SVM, KNN, and LR), which gives highly accurate predictions for the majority class label, which in this case the class label related to negative emotions. Also, when comparing the results of macro average and micro average for both class labels, the difference is not significant, indicating that prediction accuracies are not significantly impacted by the skewness of class distribution and are not biased to a particular class label. This suggests that the proposed wellbeing prediction model trained on BERT and labelled data using the proposed distant supervision method can give accurate wellbeing predictions for Twitter chatter data.

### Evaluating the utility of the proposed framework in evaluating public wellbeing

To validate the utility of the proposed method in assessing public wellbeing in a particular context, a UAE-specific dataset was generated and targeted around COVID-19 to ensure the richness of wellbeing indicators. We generated the first bilingual (Arabic and English) geotagged, COVID-19 specific dataset that is focused on tweets generated from various locations in the UAE. The evaluation Twitter Chatter data was collected on an hourly basis using the Twitter API since October 10th, 2020, by tracking keywords related to the novel coronavirus.^1^ The English keywords used to filter the tweets are *Epidemic, Pandemic, Corona, COVID, Sterilization, Quarantine, Wuhan, Coronavirus.*

We used the proposed framework to predict wellbeing status from the public Twitter chatter data relevant to a specific sector, namely, Education. The goal is to demonstrate how online learning has affected the wellbeing of students, teachers, and parents during the pandemic and how we can support decision-makers with information to make appropriate decisions. It is apparent that the pandemic has caused disruption in all educational institutions around the world. For evaluation and illustration purposes, four timeframes were selected: (a) School Fall Semester, (b) School Winter break, (c) School Spring Semester, and (d) School Spring Break.

**Fig. 8:**
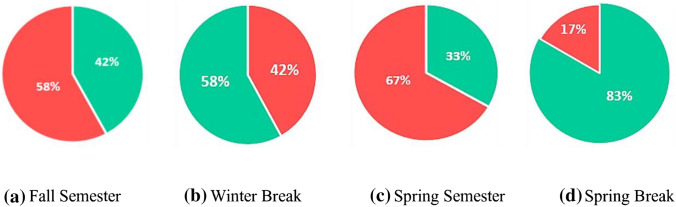
Overall sentiment score across the UAE from fall semester 2020 to spring break 2021

Figure 8 illustrates the positive to negative overall scores for each of the defined timeframes in pie charts. The red colour indicates negative emotions, while the green color indicates positive emotions. Figure 8a and c show a negative majority in the analyzed tweets, which happen to be during the first two semesters of the educational institutions’ closure and the migration to e-learning (i.e., Fall Semester 2020 and Spring Semester 2020). On the other hand, Fig. 8b and d show a positive majority during the breaks. Moreover, we can see an increased negativity score in the Spring semester compared to the Fall semester. In the fall semester, some schools managed to support a hybrid delivery mode combining face-to-face and online learning, whereas, in the Spring semester, a full lockdown was forced across the country, except in Dubai, resulting in increased overall negative emotions.

**Fig. 9:**
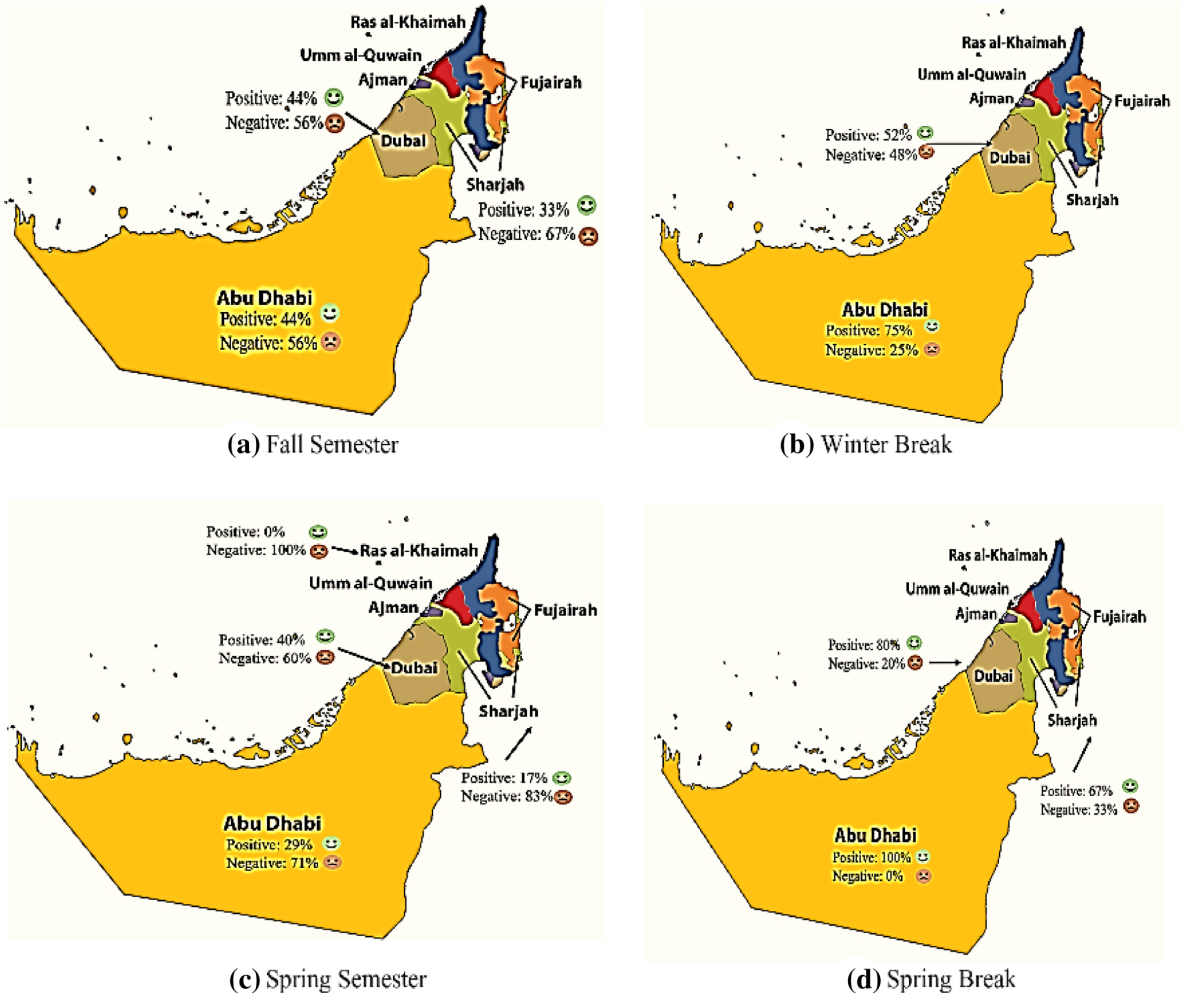
Spatial emotion distribution across the UAE

Figure 9 provides a visualization of the positive and negative emotion distribution across the different emirates of the UAE. Map visualization provides spatial analytics of emotions across several emirates. In Fig. 9, we can see the change of emotion across the emirates of Abu Dhabi, Dubai, Sharjah, and Ras al-Khaimah during the four defined timeframes. It is obvious that negativity had increased significantly in all the emirates but specifically in the emirates of Abu Dhabi, Sharjah, and Ras Al Khaimah, upon the compulsory full school closure in the Spring semester. Dubai, however, managed to continue offering full face-to-face study in addition to the hybrid mode of study, which can be seen in the minor drop in positivity compared with the other emirates.

**Fig. 10:**
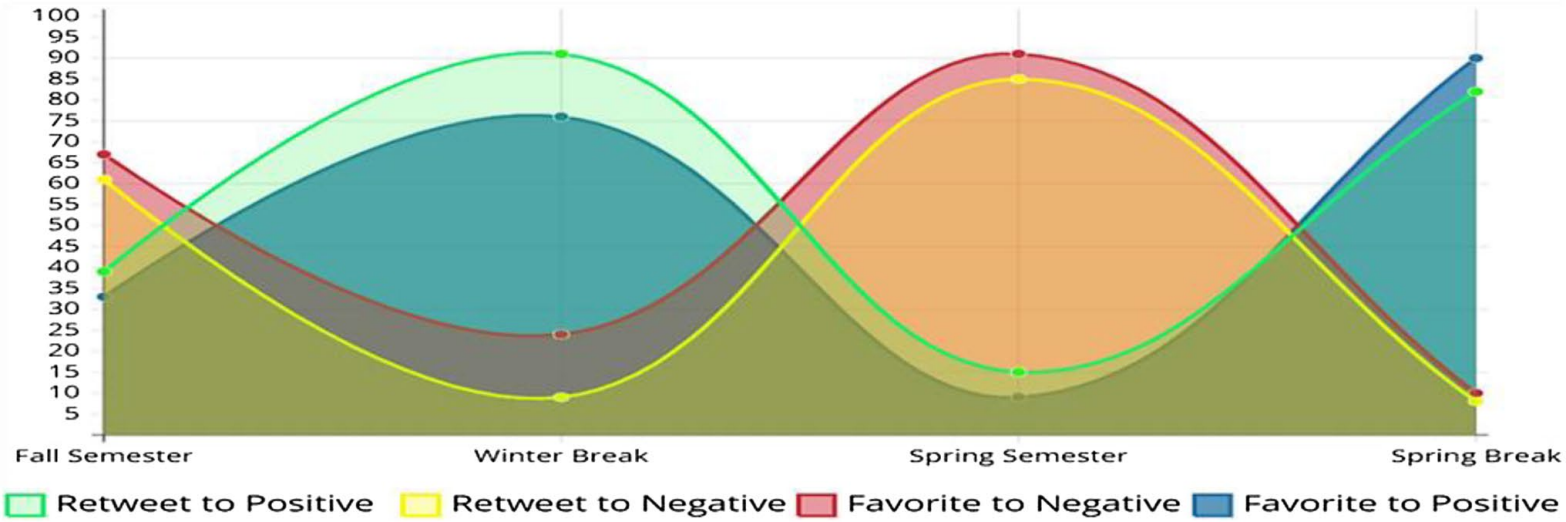
Reactions to positive versus negative tweets

In addition to the above statistics, the proposed model analyzes the interaction to positive and negative tweets given that many people might not always play an active role on social media accounts and post tweets; rather, they would just react to posted tweets. Reactions indicate people's emotions and opinions as well. Figure 10 illustrates these reactions and their change across the four defined timeframes on both positive and negative tweets. By analyzing people's reactions, we can see that the public's wellbeing indicators are consistent across the four timeframes defined for the study showing increased positivity during breaks and increased negativity during semesters with increased reactions to negative tweets during the compulsory lockdown period During Semester 2020.

As illustrated above, social media can give insights into the public wellbeing status which can support decision-makers in the decision-making process. Using this framework, governments can gauge public opinion towards specific events. For instance, by defining specific temporal events such as the first introduction of a new online examination framework', or the rectification of new rules in the education sector, officials can gauge the public's reaction to these new decisions and whether a change is required.

## Conclusion

The public wellbeing situation is directly and significantly impacted by government decisions around the globe. With the ever-changing situations and pace of modern life, public wellbeing is prioritized. Health authorities find themselves obliged to analyze the wellbeing of the public and have discovered that social media platforms are very rich sources of data that are full of emotions and wellbeing indicators. Therefore, a solution that relies on analyzing massive and spatiotemporal social media data with a focus on wellbeing will provide impactful insights.

In this paper, an intelligent decision support system is proposed encompassing several modules. The proposed system infers spatiotemporal insights on the public’s wellbeing from social media content through the implementation of a novel distant supervision approach as well as an emotion prediction model trained on semantically rich sentence embedding using BERT. Several experiments are conducted to validate the effectiveness of the proposed distant supervision approach, the prediction model, as well as the usefulness of the generated insights in supporting decision making. A further extension to the proposed framework would consider analyzing emotional triggers causing several negative and positive emotions in different sectors.

## Footnotes

1 https://github.com/RamiAltai/TwitterData
